# Trajectories of Loneliness and Psychosocial Functioning

**DOI:** 10.3389/fpsyg.2021.689913

**Published:** 2021-06-30

**Authors:** Elody Hutten, Ellen M. M. Jongen, Peter Verboon, Arjan E. R. Bos, Sanny Smeekens, Antonius H. N. Cillessen

**Affiliations:** ^1^Faculty of Psychology, Open University of the Netherlands, Heerlen, Netherlands; ^2^Pro Persona, Arnhem, Netherlands; ^3^Behavioural Science Institute, Radboud University, Nijmegen, Netherlands

**Keywords:** loneliness, trajectories, depression, anxiety, self-esteem

## Abstract

The present study examined the relationship between developmental patterns of loneliness and psychosocial functioning among adolescents (9–21 years; *N* = 110, 52% male). Four-wave longitudinal data were obtained from the Nijmegen Longitudinal Study (NLS) on Infant and Child Development. Loneliness was measured at 9, 13, 16, and 21 years of age and anxiety, depression and self-esteem at 9 and 21 years of age. Using k-means cluster analysis, three trajectories of loneliness were identified as “stable low” (56% of the subjects), “high decreasing” (22% of the subjects), and “low increasing” (22% of the subjects). Importantly, trajectories of loneliness across adolescence significantly predicted psychosocial functioning in young adulthood. Both the “high-decreasing” and “low-increasing” loneliness clusters were associated with higher risk of depression and lower self-esteem compared to the “stable low” loneliness cluster. The “low-increasing” loneliness cluster was associated with higher risk of anxiety compared to the “stable low” loneliness cluster. These results indicate that loneliness in adolescence is a vulnerability that manifests itself in higher levels of anxiety and depression and lower self-esteem in young adulthood.

## Introduction

Interpersonal wellbeing is a fundamental human need (Perlman and Peplau, 1981). The painful feeling associated with a perceived deficiency in the quantity or quality of one’s social relationships is called loneliness (Perlman and Peplau, 1981). Although loneliness is prevalent across the life span (e.g., Luhmann and Hawkley, 2016), adolescence is a period of life that is particularly interesting for studies on loneliness.

The neurological and developmental changes that characterize adolescence have been argued to put adolescents at risk of loneliness (Laursen and Hartl, 2013; Wong et al., 2018). From a life-span perspective, adolescence is marked by unique developmental challenges that change the adolescent’s social world (Erikson, 1959; Laursen and Bukowski, 1997; Laursen and Hartl, 2013). Adolescents who are unable to adapt to these changes might become lonely. According to the developmental neuroscience perspective, the changes occurring in the adolescent’s social brain make them vulnerable to developing loneliness (Wong et al., 2018). Although the prevalence of loneliness among adolescents is well established (e.g., Qualter et al., 2015), less is known about the developmental patterns of loneliness across adolescence.

Studies on loneliness are important because loneliness can harm psychosocial functioning. Previous studies have shown that loneliness is associated with anxiety, depression, and lower self-esteem (e.g., Heinrich and Gullone, 2006). According to the evolutionary theory of loneliness, feelings of loneliness can trigger a hypervigilance toward social threat (Cacioppo and Hawkley, 2009; Cacioppo and Cacioppo, 2018). This self-protective focus on threat causes cognitive biases that enforce feelings of loneliness (Cacioppo et al., 2006; Cacioppo and Hawkley, 2009). This self-reinforcing loop of loneliness can harm psychological wellbeing (Cacioppo and Cacioppo, 2018) and has been associated with psychosocial functioning (Cacioppo et al., 2006; Meng et al., 2020).

Although the association between loneliness and psychosocial functioning is well established (e.g., Heinrich and Gullone, 2006), less is known about the predictive value of developmental patterns of loneliness for psychosocial functioning. The present study explores the trajectories of loneliness from middle childhood to young adulthood and the predictive value of loneliness trajectories for psychosocial functioning in young adulthood.

Adolescence and young adulthood are periods marked by social transition. As adolescents strive for autonomy and independence, they distance themselves from their parents (Smetana et al., 2006) and peer relationships become more important (Rubin et al., 2006). Spending less time with family members and more time with peers is considered normative for this age group (Collins and Laursen, 2004). Adolescents that are unable to form close peer bonds do not conform with these age-related norms and might experience feelings of loneliness (Laursen and Hartl, 2013). Furthermore, the focus on peer relationships that characterizes adolescence can amplify feelings of difference and cause loneliness (Schinka et al., 2013). Finally, adolescence and the transition into young adulthood are characterized by identity exploration (Arnett, 2000; Laursen and Hartl, 2013; Arnett et al., 2014). This developmental process results in social instability which can lead to feelings of loneliness. As the ability to successfully navigate these changes in the social world differs between individuals, heterogeneity in the development of loneliness might occur.

Furthermore, changes occurring in the adolescent brain have been argued to put them at risk of developing loneliness according to the developmental neuroscience perspective (Wong et al., 2018). More specifically, developments of the adolescent’s social brain have been associated with increased sensitivity to the social environment and an increased vulnerability to loneliness (Blakemore, 2008).

Although cross-sectional studies have revealed an U-shaped age distribution of loneliness during the life span with elevated levels of loneliness in adolescence and old age, a recent meta-analysis of longitudinal data concluded that mean-level loneliness remains stable from adolescence to old age (Mund et al., 2020). However, this conclusion does not preclude heterogeneity in the development of loneliness. Indeed, recent studies revealed distinct trajectories of loneliness from middle childhood to young adulthood (Qualter et al., 2013; Schinka et al., 2013; Vanhalst et al., 2013a; Eccles et al., 2020).

These studies have reported three to six distinct developmental patterns of loneliness characterized by different mean loneliness levels and directions of change. Furthermore, these studies have consistently revealed that the largest portion of individuals experience stable and low levels of loneliness. However, the age ranges of the samples differed across these studies and their samples did not span the entire period of adolescence. The covered periods are early adolescence (11–15 years; Eccles et al., 2020), childhood to early adolescence (9–15 years; Schinka et al., 2013), childhood to middle adolescents (7–17 years old; Qualter et al., 2013), and middle to late adolescence (15–20 years; Vanhalst et al., 2013a). Hence, comprehensive studies exploring the trajectories of loneliness from preadolescence to young adulthood are needed (Danneel et al., 2020).

Previous studies have established an association between loneliness and psychosocial functioning in adolescence. Loneliness and depression are related but distinct concepts (Weeks et al., 1980). Depression is characterized by a loss of positive affect which manifests itself in a range of symptoms, such as loss of interest and pleasure in activities, depressogenic thoughts and sleep disturbance (American Psychiatric Association, 2013). Loneliness has been found to be a risk factor for depression in childhood and adolescence (Ebesutani et al., 2015). Anxiety is a long-term trait characterized by a non-adaptive hypervigilance and overestimation of the potential for threat in uncertain situations (Sylvers et al., 2011). Recent studies have revealed that loneliness is associated with anxiety in adolescence (e.g., Heinrich and Gullone, 2006; Ostovar et al., 2016). Anxiety has been found to be a risk factor for loneliness in childhood and adolescence (Ebesutani et al., 2015). Self-esteem refers to a favorable or unfavorable attitude toward the self and one’s worth as a person (Rosenberg, 1965). Research has revealed that self-esteem declines in adolescence (Robins and Trzesniewski, 2005; Orth et al., 2012) and is associated with anxiety, depression, eating disorders (Bos et al., 2010), and loneliness (Heinrich and Gullone, 2006; Vanhalst et al., 2013b; Geukens et al., 2020) among adolescents.

Although the association between loneliness and psychosocial functioning in adolescence is well-established, less is known about the relationship between developmental patterns of loneliness and depression, anxiety, and self-esteem. A number of studies have found an association between loneliness trajectories and psychosocial functioning (Qualter et al., 2013; Schinka et al., 2013; Vanhalst et al., 2013a). More specifically, developmental patterns of loneliness have been found to predict depression in early (Schinka et al., 2013), middle (Qualter et al., 2013), and late adolescence (Vanhalst et al., 2013a) and anxiety and self-esteem in late adolescence (Vanhalst et al., 2013a). However, Qualter et al. (2013) and Vanhalst et al. (2013a) have not provided information on the participants’ psychosocial functioning at the first measurement wave. Hence, the predictive value of loneliness trajectories for psychosocial functioning could be due to increased susceptibility to loneliness caused by early psychosocial functioning.

The present study aimed to gain insights into developmental patterns of loneliness across adolescence and the association between trajectories of loneliness and psychosocial functioning in young adulthood. Previous studies that investigated trajectories of loneliness have not used samples that span the entire period of adolescence. The current study contributes to the literature by exploring developmental patterns of loneliness using four-wave longitudinal data obtained from a nationally representative sample of Dutch children (9–21 years). The broad age range covered important developmental changes, such as the transition from elementary school to secondary school and from secondary school to higher education or the labor market. We expected to find that the majority of adolescents had low and stable loneliness levels and that loneliness trajectories that are not characterized by stable and low levels of loneliness are associated with worse psychosocial functioning.

## Materials and Methods

### Sample

Participants were 129 children (51.8% male) from the Nijmegen Longitudinal Study (NLS) on Infant and Child Development (Van Bakel and Riksen-Walraven, 2002). The NLS started in 1998 with a community-based sample of 129 infants at 15 months of age representative of the Dutch population. The sample contains children with different backgrounds in terms of family composition (e.g., single- and two-parent families, and number of siblings), parental educational attainment, characteristics of the primary caregiver (gender and employment status), and birth order (i.e., first- and later-born). The current study used data from the fifth (at the age of 9), seventh (age 13), ninth (age 16), and eleventh (age 21) wave of the NLS. The final sample contained 110 respondents.

### Measures

The measures for loneliness and psychosocial functioning were included in the study as averaged items scores. The averaged items scores were calculated for participants with a maximum of two missing item scores per measure.

#### Loneliness

At age 9, loneliness was measured with the Loneliness and Social Dissatisfaction Questionnaire (LSDQ; Asher and Wheeler, 1985). The LSDQ consists of 24 items, 16 primary items that measure children’s feelings of loneliness (e.g., “I have no one to talk to in class”) and 8 filler items about various topics aimed to help children feel more relaxed (e.g., “I watch TV a lot”). The items were rated on a five-point rating scale ranging from untrue (1) to always true (5). At age 13 and 16, loneliness was measured using a subscale of the Louvain Loneliness Scale for Children and Adolescents (LLCA; Marcoen et al., 1987). This subscale contained 12 items describing feelings and thoughts of peer-related loneliness (e.g., “I think I have fewer friends than others”). Participants rated the items on a 4-point rating scale ranging from never (0) to often (3). At age 21, participants completed the Roberts UCLA Loneliness Scale (RULS; Russell et al., 1980). The RULS consists of 20 items (e.g., “I do not feel alone”) which participants rated on a 5-point rating scale ranging from strongly disagree (1) to strongly agree (5). Loneliness scores of the different measures were rescaled to enable cluster analysis.

#### Psychosocial Functioning

Psychosocial functioning is defined as depression, anxiety, and self-esteem similar to Vanhalst et al. (2013a). Depression, anxiety, and self-esteem were measured at age 9 and 21. At age 9, anxiety was measured using the Revised Children’s Manifest Anxiety Scale (RCMAS; Reynolds and Richmond, 1979). Children rated six items on the level and nature of anxiety (e.g., “I worry a lot”) with two answer categories: This is true (1), or this is not true (2). At age 9, depression was measured using the Short Depression Inventory for Children (SDIC; De Wit, 1987). Children rated nine items on depressive symptoms (e.g., “I have been feeling sad lately”) with two answer categories: This is true (1), or this is not true (2). At age 21, anxiety and depressive symptoms were measured using the Symptom Checklist-90 (SCL-90; Arrindell and Ettema, 2004). Participants rated whether they had experienced each of the subscales’ symptoms during the past week on a 5-point scale ranging from strongly disagree (1) to strongly agree (5). The subscales contain 10 and 16 symptoms for anxiety (e.g., “worrying too much about things”) and depression (e.g., “feeling low in energy or slowed down”), respectively.

At age 9, self-esteem was measured using a subscale of the CBSK, which is the Dutch version of Harter’s self-esteem scale (The Self-Perception Profile for Children; Harter, 1985; Veerman et al., 1996). Participants rated six items (e.g., “I like the person I am”) on a 4-point scale ranging from never (1) to always (4). Self-esteem at age 21 was measured using the Rosenberg Self-Esteem Scale (RSES; Rosenberg, 1965; Franck et al., 2008). The scale consists of 10 items (e.g., “On the whole, I am satisfied with myself”) which participants rated on a 4-point scale ranging from strongly disagree (1) to strongly agree (4).

### Data Analysis

Loneliness trajectories were estimated using the four-wave loneliness data. Missing data of children with one or two missing values were imputed (53 or 12.1%) by a two-step procedure (Genolini et al., 2016). First, linear interpolation was applied (i.e., missing values were replaced with the average of non-missing, adjacent values). Second, a value was added to this average to make the imputed value follow the shape of the average trajectory.

A cluster analysis for longitudinal data was performed to extract clusters. The R package “kml” (Genolini et al., 2015) was used for the cluster analysis of the trajectories. The procedure to extract the clusters in kml is based on the classic k-means algorithm. The kml function was used with all the default options, and two to four clusters were examined. To reduce the probability of obtaining a cluster solution based on a local minimum, 20 different starting values were applied. Based on the eight fit criteria computed for each number of clusters (see for the details Genolini et al., 2015), in combination with the theoretical plausibility of the interpretation of the cluster structure, we selected the number of clusters that was used in subsequent analyses. The cluster classification was added to the data after the optimal number of clusters was selected.

Subsequently, three regression models were tested to investigate the predictive value of developmental patterns of loneliness for psychosocial functioning. The cluster classification was included as predictor for anxiety, depression, and self-esteem at age 21 while controlling for psychosocial functioning at age 9. All analyses were conducted in R (version 3.5.1).

## Results

Table 1 contains the descriptive statistics and correlations of both loneliness and psychosocial functioning at the four measurement waves.

**Table 1:** Descriptive statistics and correlations of loneliness and psychosocial functioning at different measurement waves.

S. No.		*N*	*M*	*SD*	1	2	3	4	5	6	7	8	9	10	11
1.	Loneliness 9y	110	1.71	0.49	1										
2.	Loneliness 13y	110	1.63	0.58	0.44^**^	1									
3.	Loneliness 16y	110	1.48	0.45	0.31^**^	0.41^**^	1								
4.	Loneliness 21y	110	1.65	0.56	0.30^**^	0.20^*^	0.32^**^	1							
5.	Anxiety 9y	94	1.18	0.26	0.40^**^	0.11	0.04	0.10	1						
6.	Anxiety 21y	95	1.34	0.47	0.21^*^	0.18	0.19	0.34^**^	0.17	1					
7.	Depression 9y	95	1.19	0.26	0.36^**^	0.18	0.12	0.06	0.74^**^	0.21	1				
8.	Depression 21y	95	1.48	0.48	0.07	0.22^*^	0.13	0.54^**^	0.10	0.55^**^	0.18	1			
9.	Self-esteem 9y	95	3.30	0.42	−0.32^**^	−0.41^**^	−0.26^*^	−0.15	−0.28^*^	−0.18	−0.35^**^	−0.25^*^	1		
10.	Self-esteem 21y	95	3.21	0.48	−0.13	−0.14	−0.22^*^	−0.58^**^	−0.13	−0.36^**^	−0.17	−0.51^**^	0.21	1	
11.	Gender	97			−0.15	−0.04	0.09	−0.03	−0.03	0.20	−0.02	0.19	−0.17	−0.25^*^	1

** *p* < 0.01;

* *p* < 0.05.

### Cluster Analysis

**Figure 1:**
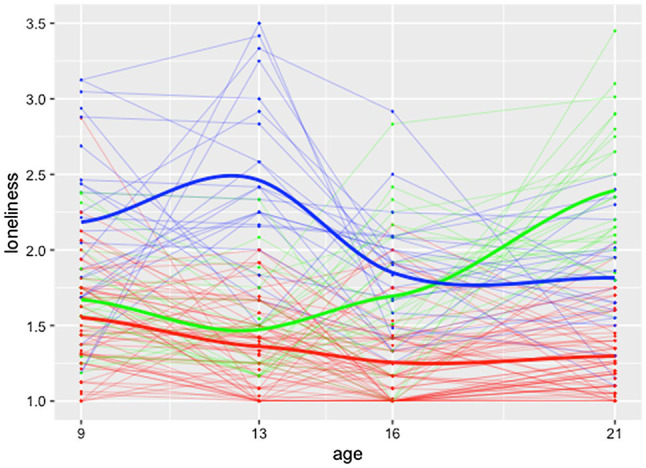
Five quality criteria for the two-, three-, and four-cluster solution as computed by the kml method.

The kml method computed five of the eight quality criteria (the other three are each similar to one of these) for the two-, three-, and four-cluster solution of the loneliness data, scaled to be between 0 and 1 (Figure 1). The criteria did not converge to the same solution. The three-cluster solution had high values for three criteria (2, 4, and 5) and medium values for the other two criteria (1 and 3). The two-cluster solution also had high values for three criteria (1, 2, and 3) but had low values for the other two criteria (4 and 5). The four-cluster solution had a high value for one criterion (5), a medium value for another criterion (4), and low values for the other three criteria (1, 2, and 3). The two- and four-cluster solutions appear suboptimal because they had criteria with scores near zero. Overall, the criteria seem to prefer the three-cluster solution.

**Figure 2:**
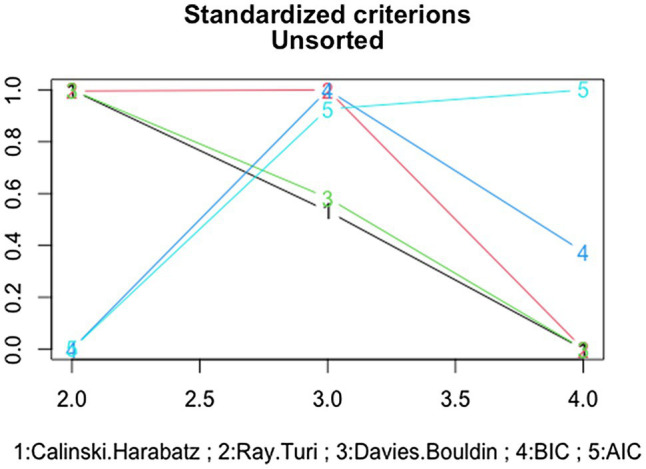
Three-cluster solution from kml. The first cluster of subjects (colored red) contains the majority (56%) of the subjects (*n* = 62) and can be described as “stable low.” The second cluster (colored blue) contains 22% of the subjects (*n* = 24) and can be described as “high decreasing.” Finally, the third cluster (colored green) contains 22% of the subjects (*n* = 24) and can be described as “low increasing.”

The three-cluster solution for the loneliness data is presented in Figure 2. Smooth curves are added to the figure to highlight the cluster trajectories. The first loneliness cluster is called “stable low” and contains the majority (56%) of the subjects (*n* = 62). This cluster is characterized by a relatively low level of loneliness from middle childhood to young adulthood. The second loneliness cluster is called “low increasing” and contains 22% of the subjects (*n* = 24). This cluster is characterized by about average loneliness scores at age 9 and higher values at the end of the measurement period (about age 21). Finally, the third loneliness cluster is called “high decreasing” and contains 22% of the subjects (*n* = 24). This cluster shows an increase in loneliness around age 13 (wave 2) and a drop after that. A chi-square test of independence showed that there was no significant association between gender and cluster membership [χ^2^ (2) = 2.93, *p* = 0.40].

To quantify the differences between the trajectories, two statistics discussed in Leffondré et al. (2004) were computed for each cluster (Table 2): (1) the standard deviation of the loneliness scores across the four waves per person, averaged over the persons within the cluster (ASD) and (2) the standard deviation of the first-order changes in loneliness across the four waves per person, averaged over the persons within the cluster (DIF). A high value of the “ASD” measure indicates that the scores are not constant (stable) over time. A high value of the “DIF” measure indicates that the first-order differences are not constant and that the pattern is therefore not linear. The results for the clusters of loneliness indicate that persons in the first cluster (“stable low”) are indeed relatively stable in loneliness scores, and persons in the second (“low-increasing”) and third (“high-decreasing”) clusters exhibit nonlinear trajectories, which is in line with the shape of the patterns as shown in Figure 2.

**Table 2:** ASD and DIF measures for the three clusters (Leffondré et al., 2004).

Clusters	ASD	DIF
Stable low	0.27	0.38
Low increasing	0.53	0.75
High decreasing	0.49	0.76

To test whether the clusters could explain variation in the loneliness scores, a multilevel analysis was conducted with the cluster index as predictor and loneliness as dependent variable. This model assumed that the scores are grouped within the four waves. The cluster index appears to be a significant predictor of loneliness [*t*(435) = 14.6, *p* < 0.001]. The intraclass correlation (ICC) with respect to the waves is 0.043. This implies that 4.3% of the variation in loneliness is explained by the four waves.

### Regression Analysis

The results of the regression analysis with depression at age 21 as the dependent variable are presented in Table 3. Both the “high-decreasing” and “low-increasing” loneliness clusters were associated with higher risk of depression at age 21 while controlling for feelings of depression at age 9. The results of the regression analysis with anxiety at age 21 as the dependent variable are presented in Table 4. The “low-increasing” loneliness cluster was associated with higher risk of anxiety at age 21 while controlling for anxiety at age 9. The results of the regression analysis with self-esteem at age 21 as the dependent variable are presented in Table 5. Both the “high-decreasing” and “low-increasing” loneliness clusters were associated with lower self-esteem at age 21 while controlling for self-esteem at age 9. Finally, gender differences were found with respect to psychosocial functioning at age 21. More specifically, being female was associated with higher depression and anxiety, and lower self-esteem.

**Table 3:** Results of the regression analysis predicting depression at age 21.

	Depression 21y	
	B	SE
Loneliness trajectories		
Low increasing	0.41^**^	0.11
High decreasing	0.43^**^	0.14
Depression 9y	0.25	0.22
Being female	0.21^*^	0.09
*N*	80	
*R*^2^	0.26	
Adj. *R*^2^	0.22	

** *p* < 0.01;

* *p* < 0.05.

**Table 4:** Results of the regression analysis predicting anxiety at age 21.

	Anxiety 21y	
	B	SE
Loneliness trajectories		
Low increasing	0.16	0.11
High decreasing	0.41^**^	0.14
Anxiety 9y	0.24	0.21
Being female	0.22^*^	0.10
*N*	80	
*R*^2^	0.18	
Adj. *R*^2^	0.13	

** *p* < 0.01;

* *p* < 0.05.

**Table 5:** Results of the regression analysis predicting self-esteem at age 21.

	Self-esteem 21y	
	B	SE
Loneliness trajectories		
Low increasing	−0.56^**^	0.09
High decreasing	−0.44^**^	0.13
Self-esteem 9y	0.08	0.11
Being female	−0.30^**^	0.08
*N*	81	
*R*^2^	0.44	
Adj. *R*^2^	0.41	

** *p* < 0.01.

## Discussion

According to the evolutionary theory of loneliness, the adaptive functions of loneliness that promote short-term survival can harm psychosocial functioning in the long term (Cacioppo and Cacioppo, 2018; Goossens, 2018). Research on loneliness in adolescence is important because the developmental changes that characterize adolescence might increase their risk of loneliness (Laursen and Hartl, 2013; Wong et al., 2018) and harm their psychosocial functioning. Although previous studies have established an association between loneliness and psychosocial functioning in adolescence (e.g., Heinrich and Gullone, 2006), less is known about the relationship between loneliness trajectories and psychosocial functioning.

The present study aimed to investigate the trajectories of loneliness from middle childhood to young adulthood and their predictive value for psychosocial functioning in young adulthood. Developmental patterns of loneliness from 9 to 21 years were examined using four-wave longitudinal data from a nationally representative sample of Dutch adolescents. Furthermore, the association between the development of loneliness across adolescence and psychosocial functioning in young adulthood was explored.

### Trajectories of Loneliness

The cluster analysis revealed heterogeneity in the development of loneliness from middle childhood to young adulthood in line with previous studies (Ladd and Ettekal, 2013; Qualter et al., 2013; Schinka et al., 2013; Vanhalst et al., 2013a; Eccles et al., 2020). More specifically, three distinct clusters of loneliness were identified. Consistent with previous studies (Ladd and Ettekal, 2013; Qualter et al., 2013; Schinka et al., 2013; Vanhalst et al., 2013a; Eccles et al., 2020), most adolescents (56%) had stable and low levels of loneliness across adolescence. This result indicates that most adolescents are able to successfully navigate the developmental challenges that characterize adolescence (Schinka et al., 2013; Vanhalst et al., 2013a).

Another group of adolescents showed an increase in levels of loneliness around age 13 followed by a decline in loneliness. Early adolescence occurs roughly between ages 10 and 14. During this developmental stage, adolescents strive for autonomy and independence by distancing themselves physically and emotionally from family members (Laursen and Hartl, 2013). As a consequence, adolescents might experience unmet social needs. Although some adolescents are able to substitute time spent with family members with time spent with peers (Laursen and Williams, 1997), others might experience loneliness (Laursen and Hartl, 2013). Furthermore, adolescence is characterized by identity exploration (Erikson, 1959; Marcia, 1980; Schwartz et al., 2011; Laursen and Hartl, 2013) in, among others, the interpersonal domain (McLean et al., 2014; Albarello et al., 2018). During this process of identity development, adolescents rethink and question their peer relationships which has been found to cause feelings of loneliness (Laursen and Hartl, 2013; Kaniušonytė et al., 2019).

Another explanation for the initial increase in loneliness could be the transition from primary to secondary education which occurs at age 13 in the Netherlands. Previous studies have revealed that the disruptive transition to high school is associated with an increase in loneliness (Benner et al., 2017). Furthermore, the classroom social environment, such as adolescents’ peer status (Engels et al., 2019), bullying, and victimization (Matthews et al., 2020), is related to feelings of loneliness. Adolescents who have difficulty navigating the social transition from primary to secondary education might experience an initial increase in loneliness.

A third loneliness cluster started with relatively stable loneliness levels but showed an increase in loneliness from age 16 to age 21. This period covers both late adolescence and the transition into young adulthood. During mid- and late adolescence, romantic relationships start to develop (Laursen and Hartl, 2013) and adolescents spend more time with their romantic partner (Collins et al., 2009). Hence, one explanation for the increasing levels of loneliness at age 16 might be that individuals who are not able to conform to this age-related norm experience feelings of loneliness. Another explanation for the elevated levels of loneliness around age 21 could be the heightened instability of romantic relationships that characterizes the transition from adolescence to young adulthood (Arnett, 2000; Arnett et al., 2014). As individuals continue their identity exploration in the interpersonal domain, changes in romantic relationships are more frequent during this developmental stage (Arnett et al., 2014). This instability could cause loneliness. Furthermore, previous studies have argued that the elevated loneliness levels among young adults are attributable to the transition to university or work life (Hysing et al., 2020).

### Developmental Patterns of Loneliness and Psychosocial Functioning

The development of loneliness across adolescence predicted depression, anxiety, and self-esteem in young adulthood after controlling for early psychosocial functioning. Compared to the “stable low” cluster, the “high-decreasing” and “low-increasing” clusters were associated with a higher risk of depression and lower self-esteem. The “low-increasing” cluster was associated with a higher risk of anxiety compared to the “low stable” cluster. This result is in line with previous studies that established an association between trajectories of loneliness and psychosocial functioning (Qualter et al., 2013; Schinka et al., 2013; Vanhalst et al., 2013a). The findings contribute to the literature by demonstrating that developmental patterns of loneliness across the entire period of adolescence have predictive value for psychosocial functioning in young adulthood after controlling for early psychosocial functioning.

These findings are in line with the evolutionary theory of loneliness which predicts that loneliness can harm long-term psychosocial functioning. Loneliness triggers hypervigilance toward social threat causing lonely individuals to perceive their social interactions as more negative and less satisfying (Cacioppo and Hawkley, 2009). These interactions can cause negative affect, such as sadness, anger, or fear (Fredrickson, 2004; Cacioppo and Hawkley, 2009). If such negative affect persists, it may evolve into mental health issues (Conway et al., 2013).

However, it is important to note that some studies have reported results that challenge the long-term causal relationship between loneliness and psychosocial functioning that is predicted by the evolutionary theory of loneliness. Earlier studies have revealed that loneliness co-develops with depression and social anxiety (Danneel et al., 2020) and self-esteem (Geukens et al., 2020) in adolescence. Furthermore, the relationship between loneliness and mental health problems (e.g., major depression and anxiety disorders) in adulthood has been found to be bidirectional (Nuyen et al., 2019).

These results stress the importance of interventions that effectively reduce loneliness (Masi et al., 2011). Risk factors that predispose children to follow specific trajectories of loneliness can be used for early targeted interventions (Qualter et al., 2013; Schinka et al., 2013; Vanhalst et al., 2013a). In addition, these results indicate that monitoring the development of loneliness across adolescence in clinical practice is important. Adolescents experiencing rising levels of loneliness could benefit from interventions aimed at addressing different aspects of psychological functioning.

However, the loneliness trajectories did not consistently predict psychosocial functioning in young adulthood. Although the high-decreasing trajectory was associated with higher anxiety in young adulthood, no significant relationship was found between the low-increasing trajectory and anxiety. A possible explanation for these inconsistent results is that general anxiety is conceptually not as strongly related to loneliness as social anxiety. Social anxiety is characterized by fear of negative evaluations and avoidance of social situations (La Greca and Stone, 1993), and previous research has established positive associations between loneliness and social anxiety in childhood and adolescence (Maes et al., 2019; Danneel et al., 2020).

Furthermore, the regression analyses revealed that childhood psychosocial functioning had no predictive value for psychosocial functioning in young adulthood. Earlier studies have reported that psychosocial functioning (e.g., depression and self-esteem) is correlated over time in adolescence (Schinka et al., 2013; Danneel et al., 2020; Geukens et al., 2020). The age range covered in these earlier studies was however more narrow than in the present study. The different developmental stages covered by the age range in the present study could thus be an explanation for the present results. As children go through a variety of developmental stages from 9 to 21 years of age, the predictive value of early psychosocial functioning ceases to exist. Another possible explanation for these results is the fact that different measuring instruments were used in childhood and young adulthood.

Finally, the results of the regression analyses reveal a significant association between gender and psychosocial functioning. More specifically, females had higher levels of depression and anxiety, and lower self-esteem. These findings are in line with previous studies that reported gender differences in depression (McLaughlin and King, 2015; Danneel et al., 2020), anxiety (Van Oort et al., 2009; McLaughlin and King, 2015) and self-esteem (Bachman et al., 2011; Moksnes and Espnes, 2013) in adolescence.

### Limitations and Future Directions

The present study is one of the first studies to investigate developmental trajectories of loneliness and its association with psychosocial functioning from middle childhood to young adulthood. Research on loneliness in such a broad age range was needed to cover important age-related life events, such as the transition from elementary school to secondary school and from secondary school to higher education or the labor market. However, the present study has some limitations which should be considered when interpreting the results.

First, the study’s sample size is relatively small and the number of measurement waves is limited. The clusters of loneliness would have been more robust if the sample size and the number of measurement waves were larger. Second, the large time span covered by the data (12 years) in combination with the limited number of measurement waves might have influenced the results of the cluster analysis. The timescale has been found to affect the optimal number of clusters (Piquero, 2008), and shorter time intervals between the measurement waves would have yielded more reliable results. Third, the loneliness clusters obtained by the present study are sample specific. Hence, replication of the results in other samples covering a broad age range is needed. Finally, different measures were used to assess loneliness at different ages. However, using different measures was unavoidable given the broad age range covered by the present study and the importance of age appropriate measures.

Although there is a growing body of research that has established heterogeneity in the development of loneliness across adolescence, it remains unclear why some individuals’ loneliness levels remain stable and low and others experience an increase or decline (Wong et al., 2018). Future research could explore explanations for interindividual differences in loneliness trajectories. Furthermore, one of the loneliness trajectories identified by the present study was characterized by an increase in loneliness at age 13. Adolescence is a period of life “with marked biological, cognitive, social, psychological, and academic changes” (Finkenauer et al., 2002; Robins et al., 2002; Bos et al., 2006, p. 5) that has been argued to generate more turmoil than childhood or adulthood (Resnick et al., 1997). The increased loneliness levels observed at age 13 signal that this transition into adolescence is a particularly interesting developmental stage in research on loneliness. Furthermore, the transition from primary to secondary education occurs at age 13 in Netherlands. Another loneliness trajectory was characterized by a peak in loneliness at age 21. Around this age, the transition from adolescence to adulthood occurs. Future research could further investigate the influence of such transitions on the development of loneliness.

## Data Availability Statement

The datasets presented in this article are not readily available because the data are part of the Nijmegen Longitudinal Study (NLS). Requests to access the datasets should be directed to contacts at: https://www.ru.nl/ontwikkelingspsychologie/nls/contact/contactgegevens.

## Ethics Statement

The studies involving human participants were reviewed and approved by CMO region Arnhem-Nijmegen, Netherlands. Written informed consent to participate in this study was provided by the participants’ legal guardian/next of kin.

## Author Contributions

EJ, SS, and PV designed the study. PV and EH analyzed the data. EH wrote the original draft of the manuscript. AC collected the data. AB, EJ, and PV reviewed and edited the manuscript. All authors contributed to the article and approved the submitted version.

### Conflict of Interest

The authors declare that the research was conducted in the absence of any commercial or financial relationships that could be construed as a potential conflict of interest.
